# The scale-free nature of protein sequence space

**DOI:** 10.1371/journal.pone.0200815

**Published:** 2018-08-01

**Authors:** Patrick C. F. Buchholz, Catharina Zeil, Jürgen Pleiss

**Affiliations:** Institute of Biochemistry and Technical Biochemistry, University of Stuttgart, Stuttgart, Germany; Consejo Nacional de Investigaciones Cientificas y Tecnicas, ARGENTINA

## Abstract

The sequence space of five protein superfamilies was investigated by constructing sequence networks. The nodes represent individual sequences, and two nodes are connected by an edge if the global sequence identity of two sequences exceeds a threshold. The networks were characterized by their degree distribution (number of nodes with a given number of neighbors) and by their fractal network dimension. Although the five protein families differed in sequence length, fold, and domain arrangement, their network properties were similar. The fractal network dimension D_f_ was distance-dependent: a high dimension for single and double mutants (D_f_ = 4.0), which dropped to D_f_ = 0.7–1.0 at 90% sequence identity, and increased to D_f_ = 3.5–4.5 below 70% sequence identity. The distance dependency of the network dimension is consistent with evolutionary constraints for functional proteins. While random single and double mutations often result in a functional protein, the accumulation of more than ten mutations is dominated by epistasis. The networks of the five protein families were highly inhomogeneous with few highly connected communities ("hub sequences") and a large number of smaller and less connected communities. The degree distributions followed a power-law distribution with similar scaling exponents close to 1. Because the hub sequences have a large number of functional neighbors, they are expected to be robust toward possible deleterious effects of mutations. Because of their robustness, hub sequences have the potential of high innovability, with additional mutations readily inducing new functions. Therefore, they form hotspots of evolution and are promising candidates as starting points for directed evolution experiments in biotechnology.

## Introduction

Power laws of the form f(x) ~ x^γ^ are ubiquitous in many physical systems and describe scale free phenomena, for which changing the scale of the independent variable x preserves the functional form f of the solution (f(λx) = λ^γ^ f(x)) [1]. Because scaling is a manifestation of the dynamics and geometry of a physical system, scaling laws reflect underlying generic features and provide insight into important universal principles, characterized by the scaling exponent γ.

Power laws also play an important role in life sciences. Spanning many orders of magnitude, fundamental variables such as metabolic rate, growth rate, or tree height follow a power law with an exponent γ which is an integer multiple of ¼ [2]. The observation of scaling relationships throughout the living world has inspired the search for basic principles that explain complex biological phenomena from unicellular organisms to trees. Power laws also describe population genetics for unlinked loci in the monomorphic limit and are a consequence of Darwin's theory of evolution [3]. For proteins, scaling relations were observed for the solvent-accessible surface area [4], the packing [5], and the equilibrium dynamics [6], and it has been suggested that near-criticality might be a characteristic of biological systems [7].

Power law distributions have also been detected in sequence similarity networks of proteins [8] and have been interpreted as a consequence of evolution [9,10] or the constraints of protein structure [9,11]. Detailed modelling of protein evolution is challenging due to the high complexity of combining random genotypic variation with selection of phenotypic traits such as folding pathway, protein stability, and biological function of the protein. Usually, the effects of mutations are non-additive and dominated by epistasis [12]. Moreover, only an infinitesimally small fraction of the sequence space of proteins has been inspected yet, despite the rapidly growing amount of DNA data due to advances in DNA sequencing techniques. While we currently know the sequences of 10^8^ proteins [13], the number of different protein sequences existing in the biosphere was estimated to be 10^34^, and up to 10^43^ different protein sequences might have been explored during 4 Gyr of evolution [14]. Though this number seems to be large, it is extremely small as compared to the number of possible protein sequences (10^300^ theoretical sequences for a medium-sized protein). Thus, we only know a tiny fraction of the total sequence space of viable proteins, and the theoretical sequence space is sparsely populated by the extant proteins.

In the absence of knowledge about the extant sequence space, relationships between known sequences can be measured by a metric based on global sequence identity or by neighborhood relationships in a protein sequence network where sequences form nodes that are connected by edges [15,16]. While pairwise sequence identity can be determined for all protein families, extended protein sequence networks only exist for families with high microdiversity such as TEM β-lactamases, which form a single connected network of more than 260 single point variants. In this network, the number of neighbors of each sequence is not equally distributed, but follows a power law with a scaling exponent of 1.2 [16]. The protein sequence network of TEM β-lactamases contains a few "hubs" such as TEM-1 and TEM-116 [17] and a large number of less connected nodes, with about 10 times less sequences having 10 times more neighbors each. It is tempting to relate the property of being a highly connected node to the property of being an ancestral sequence by intuitively assuming a preferential attachment model of network generation [18]. However, the observed scale-free degree distribution can result from a variety of different mechanisms [19] and might be determined by the actual constraints of the system rather than a unique mechanism [20].

A central constraint in protein evolution is the evolvability of a protein sequence, which includes two elements, robustness to faults and innovability [21]. Innovability seems to be a consequence of active site location [21]. Robustness can be measured by the tolerance of a protein for deleterious effects of mutations and is related to stability and conformational dynamics of a protein [22,23]. Thus, robustness is expected to vary between and inside a protein family, and it is desirable to identify or construct highly evolvable protein family members as promising starting points for directed evolution experiments.

## Methods

### Datasets of protein sequences

The datasets of the individual protein families were updated by performing BLAST searches against the non-redundant protein database from the NCBI (GenBank)[24]. The sequence datasets were updated for the families of TEM β-lactamases (TEM, 422 sequences), β-hydroxyacid dehydrogenases/ imine reductases (bHAD, 30781 sequences), thiamine diphosphate-dependent decarboxylases (DC, 39290 sequences), ω-transaminases (oTA, 120921 sequences), and short-chain dehydrogenases/ reductases (SDR, 141496 sequences). In case of TEM β-lactamases, the core region from positions 24 to 280 was used only (referring to TEM-1 position numbering).

### Sequence alignments and sequence networks

The distances between pairs of protein sequences can be measured either by counting point mutations or by pairwise sequence alignments. The former metric was applied for the densely connected family of TEM β-lactamases, for which a single point mutation forms the minimal distance between two sequences. TEM β-lactamase protein sequences were connected by an edge, if they differed by one point mutation, which was feasible due to the high microdiversity of this protein family.

Pairwise distances between sequences of the remaining protein superfamilies were calculated by combining the heuristic alignment approach of USEARCH, which reduced the number of sequence pairs, with global Needleman-Wunsch sequence alignment [25,26]. USEARCH alignments were performed to identify highly similar neighbor sequences with an identity threshold of 0.5, corresponding to 50% sequence identity without terminal gaps. In the second step, more accurate global sequence identities were derived from pairwise Needleman-Wunsch alignments (implemented in the EMBOSS bioinformatics software suite [27]) with gap opening penalty of 10 and gap extension penalty of 0.5. USEARCH and EMBOSS were run on multiple threads by applying GNU Parallel [28].

The point-mutation network of TEM β-lactamases and the identity-based networks of the remaining protein superfamilies, i.e. the sequence networks calculated by global sequence alignments as described above, were constructed and visualized by Cytoscape (version 3.4.0) using prefuse force directed layout. For the identity-based networks, prefuse force directed layout was applied with respect to the edge weights (i.e. the higher the sequence identity, the closer the sequences are placed).

### Degree distribution and fractal network dimension

For identity-based sequence networks, the number of neighboring sequences for a given sequence, i.e. the degree of a network node, was determined by counting the number of sequence pairs having a minimum sequence identity to the respective sequence, such as ≥95% sequence identity and thus less than 5% pairwise distance. For the point mutation network of TEM β-lactamases, the degree of a network node was determined by counting neighboring sequences within the distance of one point mutation to the respective sequence. The degrees were calculated for all sequences of a given sequence network and the number of sequences N having n neighbors was plotted over the degree n.

To derive the fractal network dimension D_f_ of identity-based sequence networks, the number of sequence pairs p(d) that differed by less than d%, with d% = (100—sequence identity)%, was computed for pairwise sequence identities determined by USEARCH [25]. The respective fractal network dimension D_f_ was calculated assuming p(d) ~ d^Df^ and plotting log(p(d)) over log(d) for d = 2, 4, 6, …, 100. In addition, p(d) was determined for the point-mutation network of TEM β-lactamases with d = 1, 2, 3, 4 point mutations.

## Results

All members of a protein family are related to each other by their global sequence identity obtained from pairwise sequence alignments. This relationship was analyzed by constructing networks where the nodes represent individual sequences and the edges represent a neighborhood relationship. Two types of neighborhood relationships were applied for network construction. In identity-based networks, an edge is formed between a pair of sequences if their global sequence identity exceeds a threshold. By adjusting the sequence identity threshold, the construction of identity-based sequence networks was feasible for all homologous protein families and resulted in connected networks for each family. In the rare case of protein families with high microdiversity, such as the TEM β-lactamase family, a second network type was constructed, where an edge between two nodes was formed if the two sequences differed by a point mutation. If such a point mutation network is feasible, it is expected to be highly similar to the respective identity-based network with high sequence identity threshold.

### Network models for TEM β-lactamases, a family of high microdiversity

**Fig 1:**
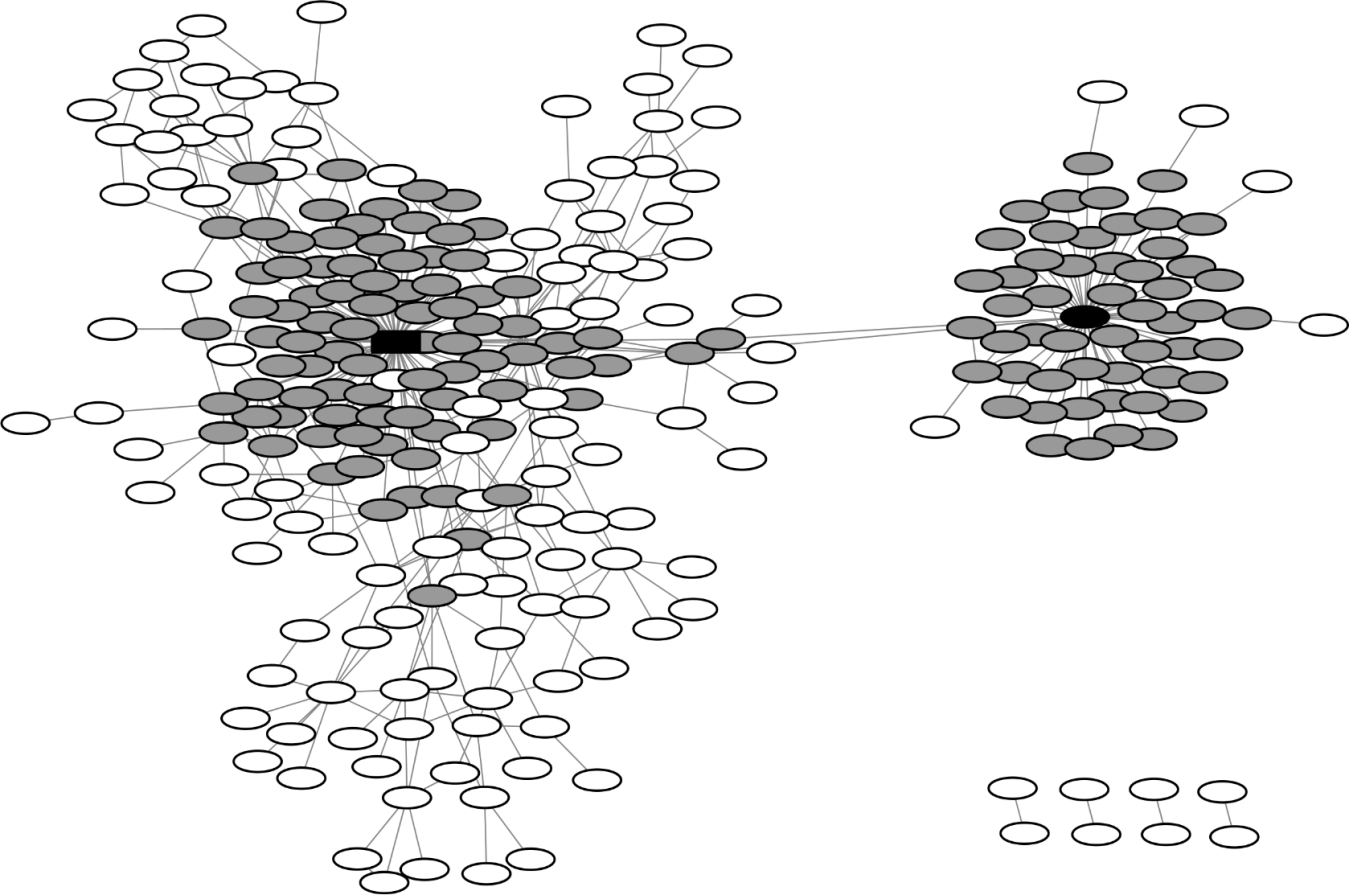
Sequence network for 267 TEM β-lactamases formed by 401 point mutations (edges) with 259 sequences forming a densely connected network with two hub sequences (TEM-1 depicted as black rectangle, TEM-116 as black oval). First neighbors of hub sequences are depicted in dark gray, other sequences in white.

**Fig 2:**
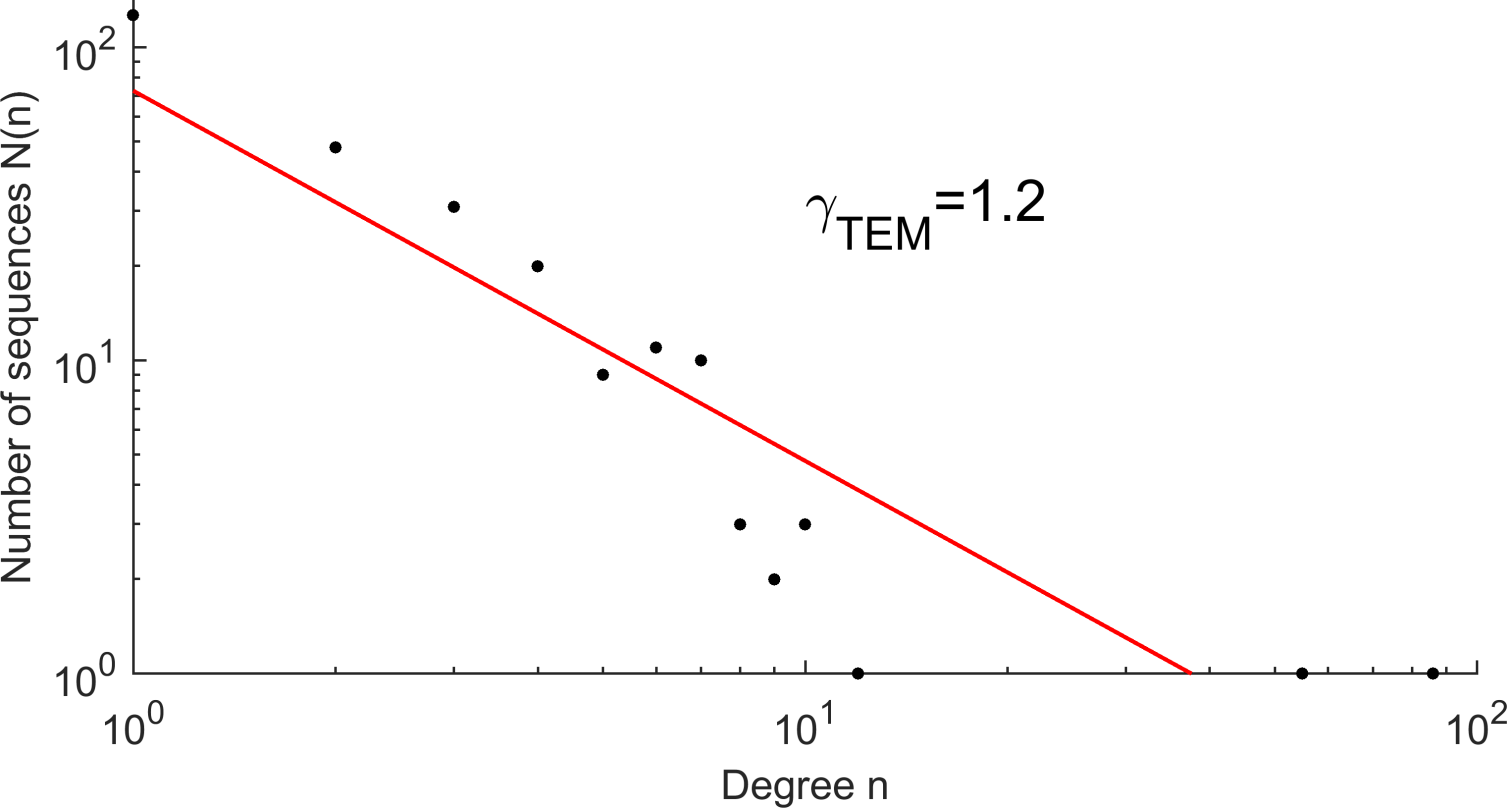
Distribution of the number of sequences N in a network of TEM β-lactamase point mutations having n first neighbors (Fig 1). The degree distribution follows a power law with exponent γ = 1.2.

The TEM β-lactamase family is a large protein family of high microdiversity [16]. A point mutation network was constructed for variants of the TEM β-lactamase core region, resulting in 267 nodes and 401 edges (**Fig 1**). The number of neighbors varied widely for each node. While there were two highly connected hubs (TEM-1 with 86 and TEM-116 with 55 neighbor sequences), most nodes had only few neighbors. The network properties were characterized by calculating the degree distribution, and the number N of nodes with n neighbors followed a power law distribution N(n)~n^-γ^ with a scaling exponent γ = 1.2 (**Fig 2**).

For comparison, an identity-based network was constructed using a global sequence identity threshold of 99.5% pairwise sequence identity, corresponding to a distance of one point mutation (**S1 Fig**). The global sequence identity measures the number of mutations between two sequences, but is independent of the number of known sequences between the two sequences. The n network consisted of 267 nodes and 401 edges, too, and its degree distribution followed a power law with a scaling exponent γ which was identical to the point mutation network (**S2 Fig**).

Alternatively, the degree distributions of the sequence networks were fitted by a Poisson distribution P(λ) and a Gaussian distribution G(μ,σ). In contrast to the power-law distribution, the Poisson and the Gaussian distribution resulted in noticeably qualitative deviations from the experimental data (**S2 Fig**).

### Degree distributions for protein superfamilies with low microdiversity

**Fig 3:**
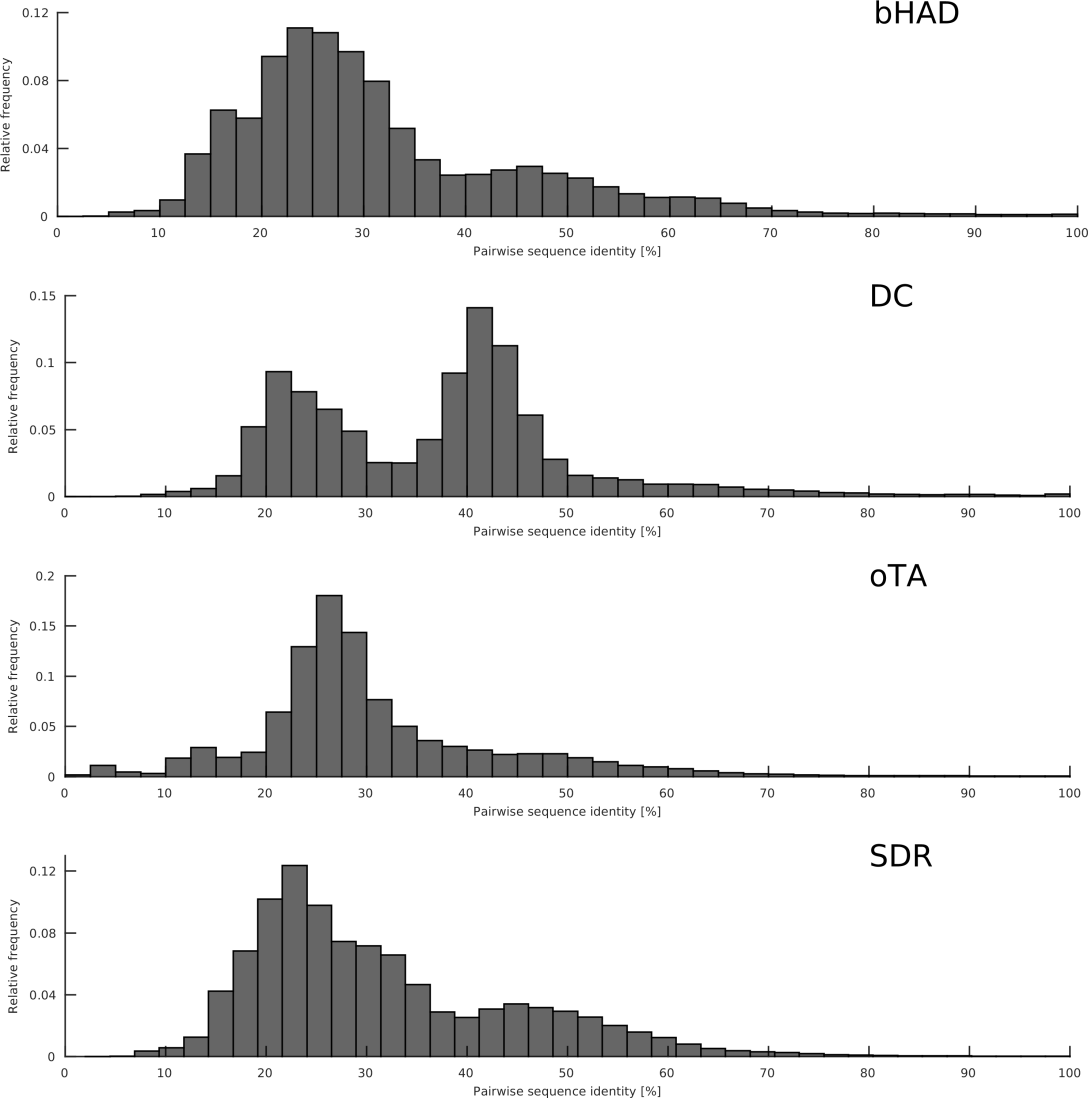
Distributions of pairwise global sequence identity for the protein families from Table 1 as determined by high-scoring sequence pairs in USEARCH (20).

Except for the TEM β-lactamases, the microdiversities of the protein families were too low to result in connected point mutation networks. Therefore, four protein superfamilies (β-hydroxyacid dehydrogenases/imine reductases, bHAD; thiamine diphosphate-dependent decarboxylases, DC; ω-transaminases, oTA; short-chain dehydrogenases/reductases, SDR) were analyzed by constructing networks based on pairwise sequence identity (**Table 1**). The protein families differed in their distributions of pairwise sequence identities, which is expected for superfamilies of different sequence length, fold, and domain arrangement (**Fig 3**).

**Table 1:** Overview of the analyzed protein family networks by number of nodes (sequences) and maximal degree (number of neighbors) for a 95% sequence identity threshold, with average sequence length.

Enzyme family (abbreviation)	Nodes	Maximal degree	Length
TEM β-lactamases (TEM)	267^a^	86^a^	250
β-hydroxyacid dehydrogenases/imine reductases (bHAD)	17020	259	320
thiamine diphosphate-dependent decarboxylases (DC)	24880	266	580
ω-transaminases (oTA)	79987	381	460
short-chain dehydrogenases/reductases (SDR)	81680	312	300

The small family of TEM β-lactamases is shown as reference due to its high microdiversity with a threshold of 99.5% sequence identity (^a^).

**Fig 4:**
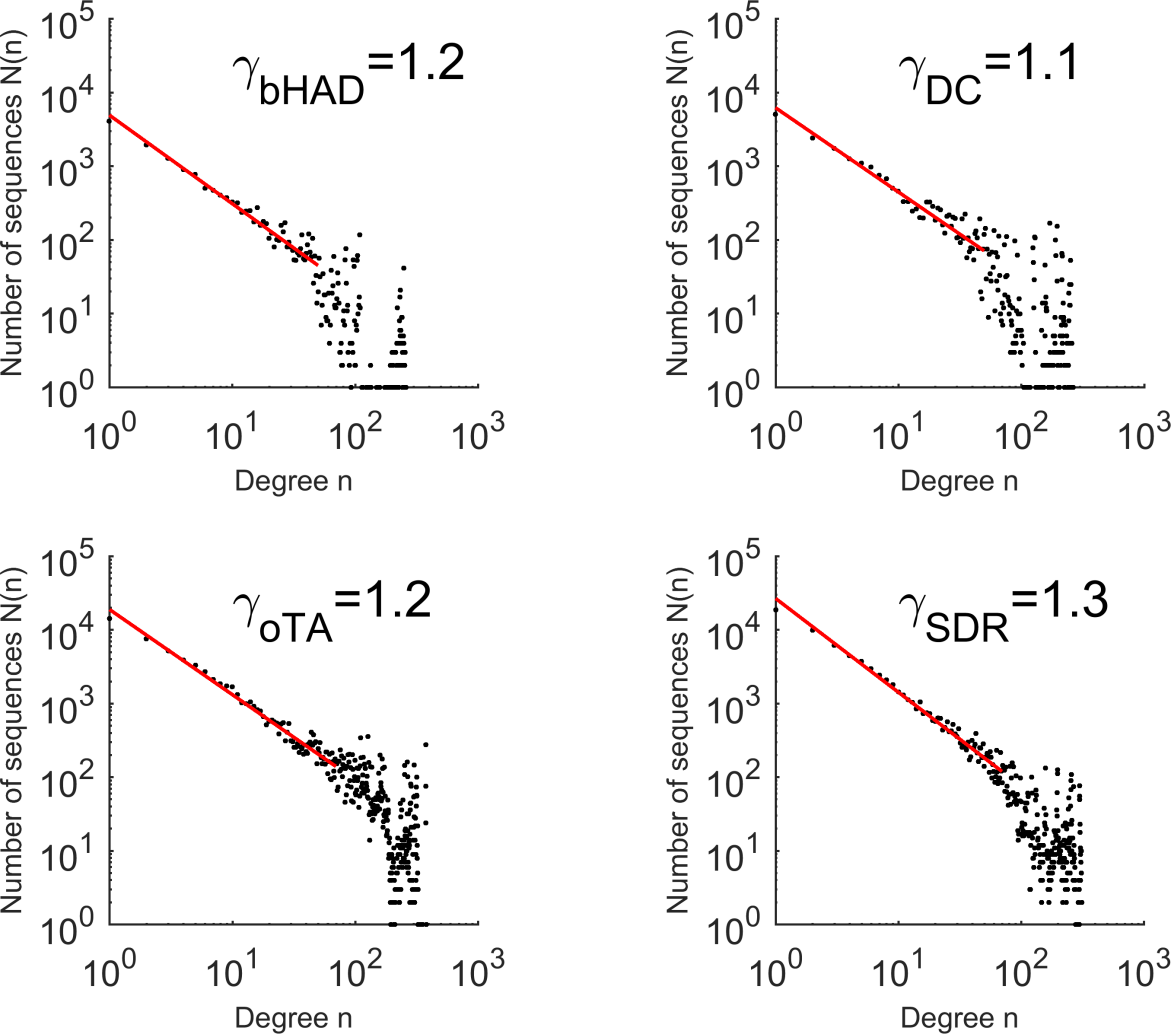
Neighbor distribution for the protein families with low microdiversity from Table 1 with neighbors defined by ≥95% global sequence identity. The corresponding scale-free exponents γ were derived from linear regression for degrees ≤ 50 (bHAD, DC) or ≤ 70 (oTA, SDR) and are summarized in **Table 2**.

Sequence pairs with a global sequence identity ≥95% were defined as neighbors. For the four identity-based networks, the degree distribution was approximated by a power law distribution N(n)~n^-γ^, whereas the distributions deviated from a scale-free behavior for the most highly connected nodes (**Fig 4**). Thus, data for degrees ≥50 or 70 were excluded from linear regression, resulting in scaling exponents of γ = 1.2–1.3 (**Table 2**). The power law distribution was maintained upon decreasing the global sequence identity thresholds for the construction of identity-based networks to ≥90%, ≥85%, or ≥80% (**S3–S5 Figs**), and the scaling exponents γ decreased slightly with decreasing threshold to γ = 0.9–1.1. Furthermore, subsets between 10% and 90% randomly selected sequences from the DC superfamily resulted in similar scaling exponents γ between 1.1 and 1.4 (**S1 Table**).

**Table 2:** Overview of the analyzed protein families from Table 1 and their derived parameters.

Enzyme family	γ	D_f_
TEM	1.2^a^	1.8
bHAD	1.2	1.0
DC	1.1	0.7
oTA	1.2	0.9
SDR	1.3	1.0

**Fig 5:**
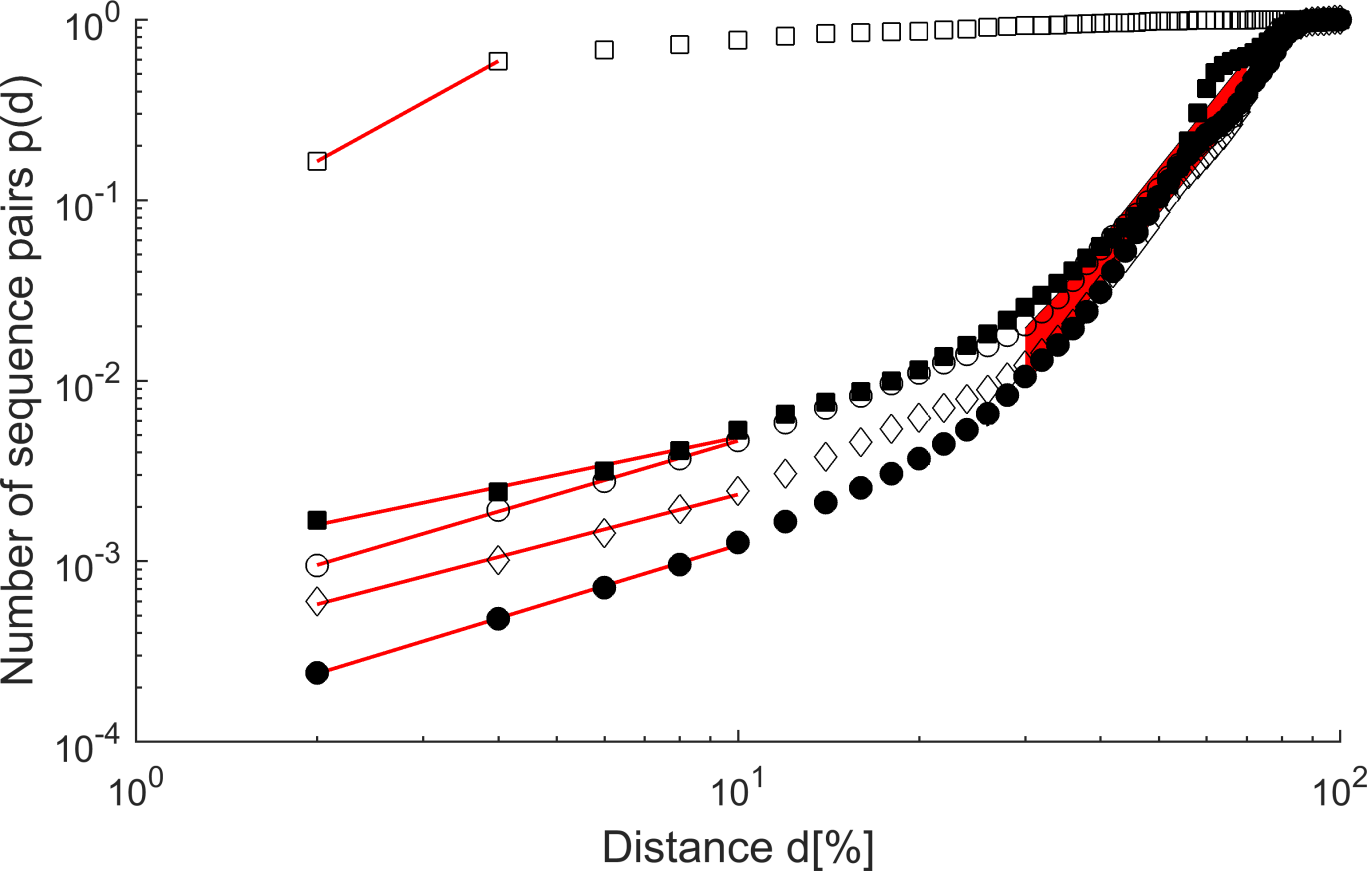
Cumulative distributions of sequence pairs p(d) for pairwise distances of d% of the protein families TEM (open squares), DC (filled squares), bHAD (open circles), SDR (diamonds) and oTA (filled circles) from Table 1 in subsequent distance intervals of 2% distance d (100%—sequence identity). Linear fits are shown as red lines for distances up to 10% identity (up to 4% for TEM). For further distances between 70 and 30%, an approximately linear area is depicted in red.

The scale-free exponent γ refers to sequence identity networks constructed with pairwise identity thresholds of 95% (compare with **Fig 4**, 99.5% threshold for TEM β-lactamases ^a^). Network dimension D_f_ refers to the slope from **Fig 5**in different regions of pairwise sequence identity (>90%).

The inhomogeneous power law degree distributions of identity-based sequence networks point to the existence of highly connected hubs in the sequence space of the four protein superfamilies (**Table 3**). Instead of individual hub sequences, communities of highly connected nodes with similar degrees were identified in the identity-based networks. For the DC superfamily, the 100 most highly connected protein sequences had between 250 and 266 neighboring sequences. Upon random selection of a subset of protein sequences from the DC superfamily, the respective sequences with the highest number of neighboring sequences were found to be highly similar, unless very small subsets were analyzed (**S2 Table**).

**Table 3:** Exemplary network hubs and their annotations from sequence networks with a threshold of 95% sequence identity (99.5% for TEM β-lactamases)^a^ for the protein families from Table 1.

Family	Annotation	Source	NCBI accession	Degree
TEM^a^	β-lactamase TEM-1	*Acinetobacter baumannii*	AAP20891	86
bHAD	2-hydroxy-3-oxopropionate reductase	*Proteobacteria*	WP_001303675	259
DC	pyruvate dehydrogenase subunit	*Gammaproteo-bacteria*	WP_044256366	266
oTA	putrescine aminotransferase	*Enterobacter cloacae*	WP_042715413	381
	aspartate aminotransferase	*Shigella*	WP_000069444	378
SDR	GDP-mannose 4,6-dehydratase	*Helicobacter pylori*	WP_058338748	312

### Dimensions of protein sequence networks

As a further network property, the fractal network dimension D_f_ was evaluated by counting the number of sequence pairs p(d) that differed by less than d% (100%—sequence identity) for d = 2, 4, 6, … (**Fig 5**). For low values of d (d≤10%, i.e. ≥90% identity), log p(d) increased linearly with log d, resulting in a network dimension D_f_ = 0.7–1.0 for the four superfamilies with low microdiversity (**Table 2**). Random selection of a subset of protein sequences from the DC superfamily lead to almost identical values for of D_f_ ≈ 0.7 for d≤10% (**S6 Fig**). For increasing distance d, the network dimension D_f_ increased to D_f_ = 3.5–4.5 for 30%≤d≤70%. For the family of TEM β-lactamases, D_f_ was estimated to 1.8 from the values at d = 2% and d = 4%. Because of the high sequence identities of the members of the TEM β-lactamase family, only few sequence pairs showed distances higher than 4% identity. Estimating the fractal network dimension for the point-mutation network of TEM β-lactamases by comparing the number of single and double mutants resulted in a higher value of D_f_ = 4.0 (**S7 Fig**). Beyond double mutants, the limited network size resulted in an apparent decrease of the network dimension, and the analysis of double, triple, and quadruple mutants resulted in D_f_ = 1.8, as observed for the identity-based TEM β-lactamase network.

## Discussion

### The dimension of protein sequence space

The evolution of protein sequences occurs in iterative steps of random mutagenesis of the genotype and subsequent selection of the phenotype. Therefore, the sequence space that has been iteratively explored during 4 Gyr of evolution is expected to be connected [29]. Since the number of explored protein sequences (10^40^) is much smaller than the number of theoretical sequences (>10^300^), the dimension of the sub-space of extant protein sequences is expected to be much smaller than the multi-thousand dimensional space of theoretical sequences. An estimation of the dimension of the known sequence space was achieved by counting the numbers of neighbors at increasing distances. The fractal network dimension D_f_ of a protein family was similar among the investigated protein families. D_f_ varied between 0.7 and 1.0 for sequence identities between 98% and 90%, whereas D_f_ increased to values between 3.5 and 4.5 at lower sequence identities between 70% and 30%. The observation of a distance-dependent fractal dimension of sequence space gives an interesting insight into the sequence-function relationships of proteins. For uncorrelated random mutations, it has been estimated that the probability of protein inactivation is 34% for each mutation [30]. Therefore, for a small number of mutations, the chance of finding active mutants is high (0.66^2^ = 44% and 0.66^4^ = 19% for two and four mutations, respectively). Thus, many combinations of random mutations result in active proteins, and D_f_ ≈ 4.0 as evaluated for the point-mutation network of TEM β-lactamases is a lower limit of the dimension of the extant sequence space for a small number of mutations, because D_f_ is expected to further increase as more TEM β-lactamase sequences are discovered in the future. In contrast, if 10% of all positions are randomly exchanged, the chance of finding an active variant of a 300 amino acid protein reduces to 0.66^30^ = 4·10^−6^. Therefore, the mutations that result in an active protein must be highly correlated, and evolution is dominated by the non-additive effects of epistasis [12]. The high correlation of mutations is compatible with the much lower fractal network dimension D_f_ = 0.7–1.0, which seems to be a generic property of all investigated protein families. For lower sequence identities between 70 and 30%, the mutations become more uncoupled, which results in a considerable increase of the fractal network dimension D_f_.

At a first glance, scale-dependent network dimensions are counter-intuitive. However, scale-dependent spatial dimensions have also been observed for physical systems such as turbulent interfaces [31] and for the distribution of luminous matter in the universe [32]. Although the analysis of the distance dependence of protein sequence space is based on a relatively small number of known sequences, it provides quantitative estimates which are in agreement with known sequence-function relationships [30]. It will be interesting to see how D_f_ develops in the future, when many more protein sequences become known.

### Evolutionary constraints for protein sequence space

Two complementary neighborhood definitions were applied to construct sequence networks. A network construction based on point mutations allows for an interpretation of alternative evolutionary paths along the network [16]. However, mutation-based networks are restricted to the rare families with high microdiversity such as TEM β-lactamases. In contrast, the metric of global sequence identity can be applied to all protein families. For TEM β-lactamases, the mutation-based and the identity-based degree distributions were identical and were approximated by a power law distribution with a scaling exponent γ = 1.2. A power law degree distribution was also observed for four protein families with low microdiversity (bHAD, DC, oTA, SDR) when using the distance metrics. Although the four families have different structural folds, domain arrangements, and sequence lengths, and differ in their level of sequence diversity (**Fig 3**) and their size (**Table 1**), they resulted in similar scaling exponents γ = 1.2–1.4. The observation that different protein families show similar scaling exponents indicates that the constraints that govern protein evolution are similar for all proteins [20].

Scale-free distributions of protein families have been described previously for networks of co-occurring protein domains and networks of sequence motifs, with scaling exponents γ in the range from 1.7 to 2.0 [11,33]. By clustering sequences into homologous families, scale-free cluster size distributions have been observed with scaling exponents between 1.6 and 2.5 [8,10,34,35]. It has been suggested that cluster size distribution is a direct consequence of the necessity for a functional protein to fold into a stable structure [9]. As a consequence, sequence space is highly connected, as seen for families with high microdiversity [16]. Connectivity is also related to findability of genotypes [36]. Stability against random errors, another feature attributed to scale-free networks, is also favorable during evolution [37].

### Pitfalls and limitations for protein sequence networks

While scale-free distributions seem to be ubiquitous in many domains of life sciences, care should be taken when drawing far-reaching conclusions which are not supported by the data [19]. Therefore, the goodness of the power law fit was compared to alternative fits by Poisson and Gaussian distributions. While the parameters of the Poisson and Gaussian distributions could be adjusted to follow the data in the tail, they fail to describe the monotonous increase of the number of nodes at decreasing degrees, and thus confirm the power law fit [19,20]. However, the limited number of sequences per protein family and the small fraction (10^−20^) of known protein sequences [14] are two factors that favor the tendency to form a power law distribution, because it has been observed that binning of the data has the tendency to form a power law distribution [38] and that sub-networks tend to exhibit a power law distribution, irrespective of the topological property of the larger network they were sampled from [39]. By analyzing randomly selected sub-networks, we demonstrated that the scaling exponent was robust upon resampling, thus excluding the possibility that the scaling exponent might differ between network and sub-networks [40]. However, there is still a risk that the apparent power law distribution might result from a sampling artefact. As the number of newly sequenced genomes is rapidly expanding in the near future, it will be interesting to see whether the degree distribution is robust upon better sampling of the sequence space.

### Implications for protein evolution and protein engineering

Protein networks with a highly inhomogeneous, exponential degree distribution with a long tail have another interesting consequence: the existence of a few highly connected nodes. These hubs are sequences or groups of sequences with a very large number of potentially functional neighbors.

The role of hubs in evolution is still under discussion. It has been suggested that highly connected nodes originated early in evolution [41], while less connected nodes are recent results from divergent evolution [42]. This interpretation of "the old get richer" is based on preferential attachment network models [18]. However, preferential attachment is only one way to generate networks, and there are different network topologies which all result in a power law degree distribution [19]. As a consequence, the most highly connected protein sequences are not necessarily the phylogenetically oldest, thus hub sequences should not be interpreted as ancestors. By assuming that evolution has reached an equilibrium in protein sequence space, the more evolvable folds might have become densely populated as a consequence of convergent evolution [42], thus connecting the concept of hubs to the concept of evolvability. The observation of a uniform distribution of sequences from thermophilic and hyperthermophilic sources in the oTA network demonstrated that hub sequences are not characterized by increased thermostability (S2 Fig in [43]).

Evolvability of a protein sequence has two aspects: robustness toward possible deleterious effects of mutations and innovability, where additional mutations readily induce new functions [21]. Since the hub sequences have many supposedly functional neighbors, they have proven to be highly evolvable. Interestingly, some hub proteins have a pivotal role in metabolism. The E1 subunit of the pyruvate dehydrogenase complex, a hub of the DC network, is also a hub in the metabolism linking glycolysis and citric acid cycle [44,45]. The aspartate aminotransferase, a hub of the oTA network, links the amino acid and the carbohydrate metabolisms [46]. These coincidences of hubs in sequence networks and metabolic networks could point at a higher robustness against mutations to preserve cellular function.

The concept of hubs can also be applied to improve the efficiency of directed evolution experiments. Directed evolution is a powerful and widely applied strategy for improving biochemical and biophysical properties of proteins by applying iterative rounds of random mutations and screening. However, multiple random mutations tend to result in inactive proteins with a probability of 92% for only six random mutations [30]. Therefore, it has been suggested to start a directed evolution experiment either from a population of neutral mutants [47] or by constructing ancestor sequences [48] which are believed to have a higher robustness and thus higher evolvability than contemporary sequences [49]. As a promising alternative, we suggest to use the hub sequences as promising starting points in directed evolution experiments and to select highly evolvable homologues directly from the pool of contemporary sequences.

## Supporting information

S1 Table Scaling exponents γ for randomly selected subnetworks of the DC superfamily, with edges formed by a threshold of 95% pairwise sequence identity. Linear regressions were performed up to a limited number of neighbors only, due to low sampling quality for higher degrees. Thus, values for γ were determined up to a maximum degree.
(PDF)

S2 Table Exemplary protein sequences found in hub regions of the DC networks for varying subsets of randomly selected sequences. The Annotations are listed as “pyruvate dihydrogen-ase subunit” (PDH), “glyoxylate carboligase” (GLX) or “acetolactase synthase 2 catalytic subunit” (ALS). Pairwise sequence identities towards the hub sequence of the complete net-work (WP_044256366) are given in the column on the right.
(PDF)

S1 Fig Sequence network for 267 TEM β-lactamases connected by 401 edges above a 99.5% pairwise sequence identity threshold, in comparison to the point mutation network (Fig 1). Hub sequences are depicted in black (TEM-1 as black rectangle, TEM-116 as black oval) with their first neighbors depicted in dark gray, other sequences in white.
(TIF)

S2 Fig Distribution of the number of sequences N having n first neighbors for the distance-based network of TEM β-lactamases (S1 Fig). The degree distribution hints at a power law distribution with exponent γ = 1.2 (**a**). In addition, probability density functions were fitted for a power-law distribution (line, γ = 1.2), a Gaussian distribution (dashed line, μ = 3.0, σ = 6.4) and a Poisson distribution (dotted line, λ = 3.0) with residual sum of squares 0.01, 0.2 and 0.1, respectively (**b-d**).
(TIF)

S3 Fig Degree distribution for the protein families with low microdiversity from Table 1 with neighbors defined by ≥ 90% global sequence identity. Linear regression was performed for degrees ≤ 50 (bHAD, DC) or ≤ 70 (oTA, SDR).
(TIF)

S4 Fig Degree distribution for the protein families with low microdiversity from Table 1 with neighbors defined by ≥ 85% global sequence identity. Linear regression was performed for degrees ≤ 50 (bHAD, DC) or ≤ 70 (oTA, SDR).
(TIF)

S5 Fig Degree distribution for the protein families with low microdiversity from Table 1 with neighbors defined by ≥ 80% global sequence identity. Linear regression was performed for degrees ≤ 50 (bHAD, DC) or ≤ 70 (oTA, SDR).
(TIF)

S6 Fig Cumulative distributions of sequence pairs p(d) for pairwise distances of d % of the DC protein superfamily for the complete data set (open squares) and 10%, 20%, …, 90% randomly selected subsets of sequences (filled squares). The areas marked in red correspond to the linear approximations from **Fig 5**.
(TIF)

S7 Fig Cumulative distribution of sequence pairs p(d) with distance in d point mutations of TEM β-lactamases (open squares). Linear fits are shown for d = 1,2 (red line) and for d = 2,3,4 point mutations (blue line).
(TIF)

## References

[1] Newman MEJ. Power laws, Pareto distributions and Zipf’s law. Contemp Phys. 2005;46: 323–351. 10.1080/00107510500052444

[2] West GB, Brown JH. Life’s universal scaling laws. Phys Today. 2004;57: 36–43. 10.1063/1.1809090

[3] Manhart M, Haldane A, Morozov A V. A universal scaling law determines time reversibility and steady state of substitutions under selection. Theor Popul Biol. 2012;82: 66–76. 10.1016/j.tpb.2012.03.007 22838027 PMC3613437

[4] Moret MA, Santana MC, Zebende GF, Pascutti PG. Self-similarity and protein compactness. Phys Rev E—Stat Nonlinear, Soft Matter Phys. 2009;80: 1–4. 10.1103/PhysRevE.80.041908 19905343

[5] Reuveni S, Granek R, Klafter J. Proteins: coexistence of stability and flexibility. Phys Rev Lett. 2008;100: 1–4. 10.1103/PhysRevLett.100.208101 18518581

[6] Tang Q-Y, Zhang Y-Y, Wang J, Wang W, Chialvo DR. Critical fluctuations in the native state of proteins. Phys Rev Lett. 2017;118: 1–5. 10.1103/PhysRevLett.118.088102 28282168

[7] Mora T, Bialek W. Are biological systems poised at criticality? J Stat Phys. 2011;144: 268–302. 10.1007/s10955-011-0229-4

[8] Enright AJ, Kunin V, Ouzounis CA. Protein families and TRIBES in genome sequence space. Nucleic Acids Res. 2003;31: 4632–4638. 10.1093/nar/gkg495 12888524 PMC169885

[9] Deeds EJ, Dokholyan N V., Shakhnovich EI. Protein evolution within a structural space. Biophys J. 2003;85: 2962–2972. 10.1016/S0006-3495(03)74716-X 14581198 PMC1303574

[10] Koonin E V., Wolf YI, Karev GP. The structure of the protein universe and genome evolution. Nature. 2002;420: 218–223. 10.1038/nature01256 12432406

[11] Wuchty S. Scale-free behavior in protein domain networks. Mol Biol Evol. 2001;18: 1694–1702. 10.1093/oxfordjournals.molbev.a003957 11504849

[12] Wu NC, Dai L, Olson CA, Lloyd-Smith JO, Sun R. Adaptation in protein fitness landscapes is facilitated by indirect paths. Elife. 2016;5 10.7554/eLife.16965 27391790 PMC4985287

[13] Consortium UniProt. UniProt: a hub for protein information. Nucleic Acids Res. 2014;43: D204–D212. 10.1093/nar/gku989 25348405 PMC4384041

[14] Dryden DTF, Thomson AR, White JH. How much of protein sequence space has been explored by life on Earth? J R Soc Interface. 2008;5: 953–956. 10.1098/rsif.2008.0085 18426772 PMC2459213

[15] Widmann M, Pleiss J. Protein variants form a system of networks: Microdiversity of IMP metallo-beta-lactamases. PLoS One. 2014;9 10.1371/journal.pone.0101813 25013948 PMC4094381

[16] Zeil C, Widmann M, Fademrecht S, Vogel C, Pleiss J. Network analysis of sequence-function relationships and exploration of sequence space of TEM beta-lactamases. Antimicrob Agents Chemother. 2016;60: 2709–2717. 10.1128/AAC.02930-15 26883706 PMC4862526

[17] Jacoby GA, Bush K. The Curious Case of TEM-116. Antimicrob Agents Chemother. 2016;60: 7000–7000. 10.1128/AAC.01777-16 28045664 PMC5075131

[18] Barabasi A-L, Albert R. Emergence of scaling in random networks. Science. 1999;286: 509–512. 10.1126/science.286.5439.509 10521342

[19] Lima-Mendez G, van Helden J. The powerful law of the power law and other myths in network biology. Mol Biosyst. 2009;5: 1482–1493. 10.1039/b908681a 20023717

[20] Keller EF. Revisiting “scale-free” networks. BioEssays. 2005;27: 1060–1068. 10.1002/bies.20294 16163729

[21] Dellus-Gur E, Toth-Petroczy A, Elias M, Tawfik DS. What makes a protein fold amenable to functional innovation? Fold polarity and stability trade-offs. J Mol Biol. Elsevier B.V.; 2013;425: 2609–2621. 10.1016/j.jmb.2013.03.033 23542341

[22] Tokuriki N, Tawfik DS. Protein dynamism and evolvability. Science. 2009;324: 203–207. 10.1126/science.1169375 19359577

[23] Dellus-Gur E, Elias M, Caselli E, Prati F, Salverda MLM, de Visser JAGM, et al Negative epistasis and evolvability in TEM-1 β-lactamase—The thin line between an enzyme’s conformational freedom and disorder. J Mol Biol. Elsevier Ltd; 2015;427: 2396–2409. 10.1016/j.jmb.2015.05.011 26004540 PMC4718737

[24] Benson DA, Cavanaugh M, Clark K, Karsch-Mizrachi I, Ostell J, Pruitt KD, et al GenBank. Nucleic Acids Res. 2017;46: D41–D47. 10.1093/nar/gkx1094 29140468 PMC5753231

[25] Edgar RC. Search and clustering orders of magnitude faster than BLAST. Bioinformatics. 2010;26: 2460–2461. 10.1093/bioinformatics/btq461 20709691

[26] Needleman SB, Wunsch CD. A general method applicable to the search for similarities in the amino acid sequence of two proteins. J Mol Biol. Elsevier; 1970;48: 443–453. 5420325 10.1016/0022-2836(70)90057-4

[27] Rice P, Longden I, Bleasby A. EMBOSS: the European Molecular Biology Open Software Suite. Trends Genet. 2000;16: 276–277. 10.1016/S0168-9525(00)02024-2 10827456

[28] Tange O. GNU parallel: the command-line power tool. login USENIX Mag. 2011;36: 42–47.

[29] Smith JM. Natural selection and the concept of a protein space. Nature. 1970;225: 563–564. 10.1038/225563a0 5411867

[30] Guo HH, Choe J, Loeb LA. Protein tolerance to random amino acid change. Proc Natl Acad Sci U S A. 2004;101: 9205–9210. 10.1073/pnas.0403255101 15197260 PMC438954

[31] Catrakis HJ, Dimotakis PE. Scale distributions and fractal dimensions in turbulence. Phys Rev Lett. 1996;77: 3795–3798. 10.1103/PhysRevLett.77.3795 10062310

[32] Bak P, Chen K. Scale dependent dimension of luminous matter in the universe. Phys Rev Lett. 2001;86: 4215–4218. 10.1103/PhysRevLett.86.4215 11328138

[33] Aziz MF, Caetano-Anollés K, Caetano-Anollés G. The early history and emergence of molecular functions and modular scale-free network behavior. Sci Rep. Nature Publishing Group; 2016;6 10.1038/srep25058 27121452 PMC4848518

[34] Orengo CA, Thornton JM. Protein families and their evolution—a structural perspective. Annu Rev Biochem. 2005;74: 867–900. 10.1146/annurev.biochem.74.082803.133029 15954844

[35] Buchholz PCF, Fademrecht S, Pleiss J. Percolation in protein sequence space. PLoS One. Public Library of Science; 2017;12 10.1371/journal.pone.0189646 29261740 PMC5738032

[36] McCandlish DM. On the findability of genotypes. Evolution (N Y). 2013;67: 2592–2603. 10.1111/evo.12128 24033169

[37] Albert R, Barabasi A-L. Statistical mechanics of complex networks. Rev Mod Phys. 2002;74: 47–97. 10.1103/RevModPhys.74.47

[38] Jeong H, Tombor B, Albert R, Oltvai ZN, Barabási A-L. The large-scale organization of metabolic networks. Nature. 2000;407: 651–654. 10.1038/35036627 11034217

[39] Han J-DJ, Bertin N, Hao T, Goldberg DS, Berriz GF, Zhang L V, et al Evidence for dynamically organized modularity in the yeast protein-protein interaction network. Nature. 2004;430: 88–93. 10.1038/nature02555 15190252

[40] Stumpf MPH, Wiuf C, May RM. Subnets of scale-free networks are not scale-free: Sampling properties of networks. Proc Natl Acad Sci. 2005;102: 4221–4224. 10.1073/pnas.0501179102 15767579 PMC555505

[41] Fell DA, Wagner A. The small world of metabolism. Nat Biotechnol. 2000;18: 1121–1122. 10.1038/81025 11062388

[42] Dokholyan N V., Shakhnovich B, Shakhnovich EI. Expanding protein universe and its origin from the biological Big Bang. Proc Natl Acad Sci. 2002;99: 14132–14136. 10.1073/pnas.202497999 12384571 PMC137849

[43] Buß O, Buchholz PCF, Gräff M, Klausmann P, Rudat J, Pleiss J. The ω-transaminase engineering database (oTAED): a navigation tool in protein sequence and structure space. Proteins Struct Funct Bioinforma. 2018;86: 566–580. 10.1002/prot.25477 29423963

[44] Gray LR, Tompkins SC, Taylor EB. Regulation of pyruvate metabolism and human disease. Cell Mol Life Sci. 2014;71: 2577–2604. 10.1007/s00018-013-1539-2 24363178 PMC4059968

[45] Zhang S, Hulver MW, McMillan RP, Cline MA, Gilbert ER. The pivotal role of pyruvate dehydrogenase kinases in metabolic flexibility. Nutr Metab. 2014;11 10.1186/1743-7075-11-10 24520982 PMC3925357

[46] Korla K, Vadlakonda L, Mitra CK. Kinetic simulation of malate-aspartate and citrate-pyruvate shuttles in association with Krebs cycle. J Biomol Struct Dyn. Taylor & Francis; 2015;33: 2390–2403. 10.1080/07391102.2014.1003603 25559761

[47] Gupta RD, Tawfik DS. Directed enzyme evolution via small and effective neutral drift libraries. Nat Methods. 2008;5: 939–942. 10.1038/nmeth.1262 18931667

[48] Merkl R, Sterner R. Ancestral protein reconstruction: techniques and applications. Biol Chem. 2016;397: 1–21. 10.1515/hsz-2015-0158 26351909

[49] Gaucher EA, Govindarajan S, Ganesh OK. Palaeotemperature trend for Precambrian life inferred from resurrected proteins. Nature. 2008;451: 704–707. 10.1038/nature06510 18256669

