# Supplementary material for: The scale-free nature of protein sequence space
Source: PLoS One. 2018 Aug 1;13(8):e0200815. doi: 10.1371/journal.pone.0200815 (PMC6070207; doi:10.1371/journal.pone.0200815)
Supplement: S1 Table — Linear regressions were performed up to a limited number of neighbors only, due to low sampling quality for higher degrees. Thus, values for γ were determined up to a maximum degree. (PDF) [file pone.0200815.s001.pdf]

S1 Table. Scaling exponents γ for randomly selected subnetworks of the DC superfamily, with edges formed by a threshold of 95% pairwise sequence identity. Linear regressions were performed up to a limited number of neighbors only, due to low sampling quality for higher degrees. Thus, values for γ were determined up to a maximum degree.

**Selection [%]**	**γ**	**Maximal degree**
100	1.1	50
90	1.2	50
80	1.2	50
70	1.2	30
60	1.1	30
50	1.2	30
40	1.1	20
30	1.2	10
20	1.2	10
10	1.4	10
