# Supplementary material for: The scale-free nature of protein sequence space
Source: PLoS One. 2018 Aug 1;13(8):e0200815. doi: 10.1371/journal.pone.0200815 (PMC6070207; doi:10.1371/journal.pone.0200815)
Supplement: S2 Table — The Annotations are listed as “pyruvate dihydrogen-ase subunit” (PDH), “glyoxylate carboligase” (GLX) or “acetolactase synthase 2 catalytic subunit” (ALS). Pairwise sequence identities towards the hub sequence of the complete net-work (WP_044256366) are given in the column on the right. (PDF) [file pone.0200815.s002.pdf]

S2 Table. Exemplary protein sequences found in hub regions of the DC networks for varying subsets of randomly selected sequences. The Annotations are listed as "pyruvate dihydrogen-ase subunit" (PDH), "glyoxylate carboligase" (GLX) or "acetolactase synthase 2 catalytic subunit" (ALS). Pairwise sequence identities towards the hub sequence of the complete net-work (WP_044256366) are given in the column on the right.

Selection [%]	Annotation	Source	NCBI accession	Degree	Identity [%]
100	PDH	*Gamma-proteobacteria*	WP_044256366	266	100.0
90	PDH	*Citrobacter sp.* MGH 55	WP_043001220	229	99.8
80	PDH	*Gamma-proteobacteria*	WP_044256366	212	100.0
70	PDH	*Citrobacter sp.* MGH 55	WP_043001220	180	99.8
60	PDH	*Entero-bacteriaceae*	WP_000815384	146	96.2
50	PDH	*Gamma-proteobacteria*	WP_044256366	133	100.0
40	PDH	*Escherichia coli*	WP_021550521	103	95.8
30	GLX	*Salmonella enterica*	WP_038390089	68	24.0
20	PDH	*Citrobacter sp.* MGH 55	WP_043001220	51	99.8
10	ALS	*Escherichia albertii*	WP_025238020	24	28.1
