# Supplementary figures and images for: The scale-free nature of protein sequence space
Source: PLoS One. 2018 Aug 1;13(8):e0200815. doi: 10.1371/journal.pone.0200815 (PMC6070207; doi:10.1371/journal.pone.0200815)

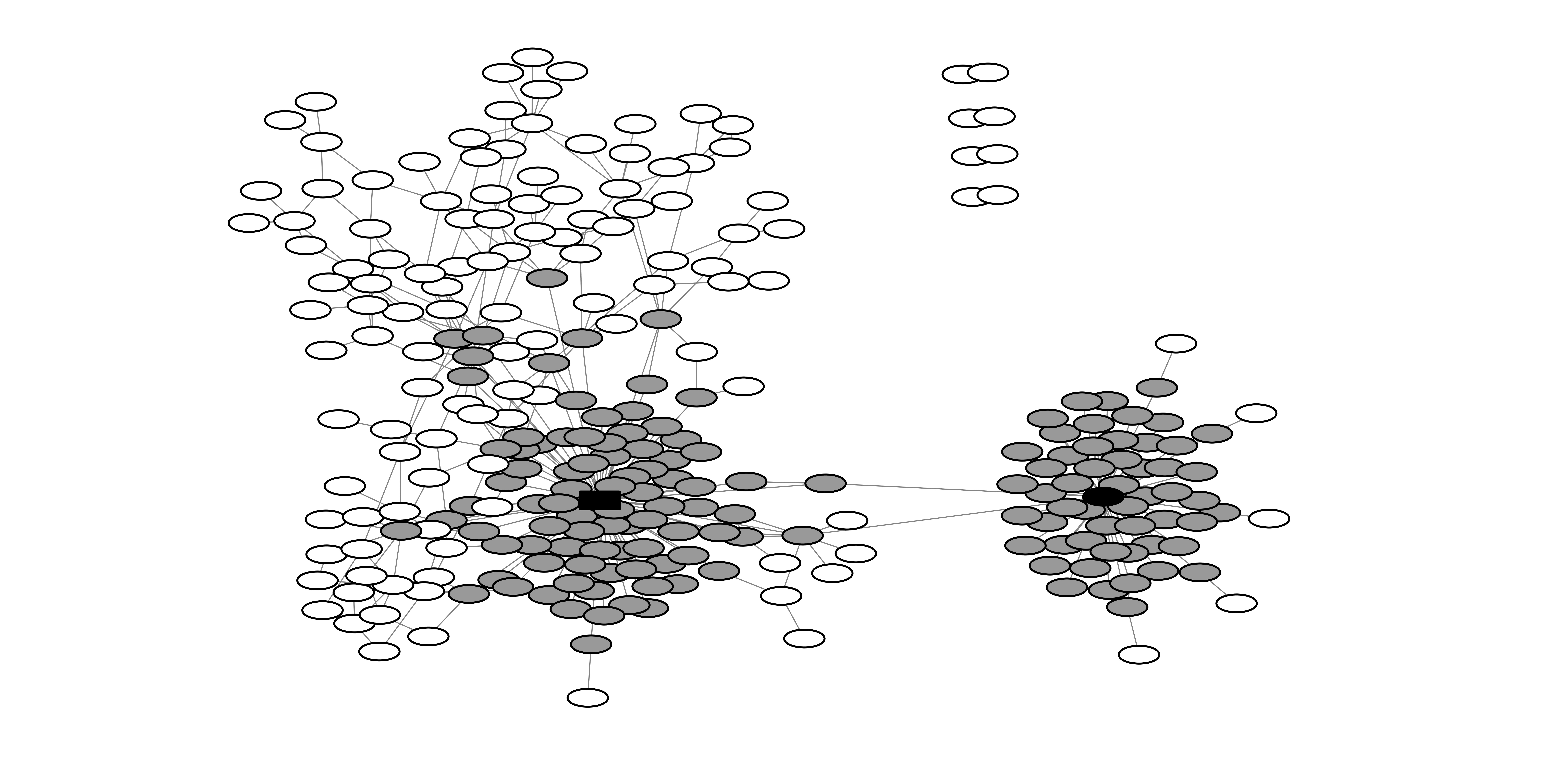

Supplement: S1 Fig — Hub sequences are depicted in black (TEM-1 as black rectangle, TEM-116 as black oval) with their first neighbors depicted in dark gray, other sequences in white. (TIF) [file pone.0200815.s003.tif]

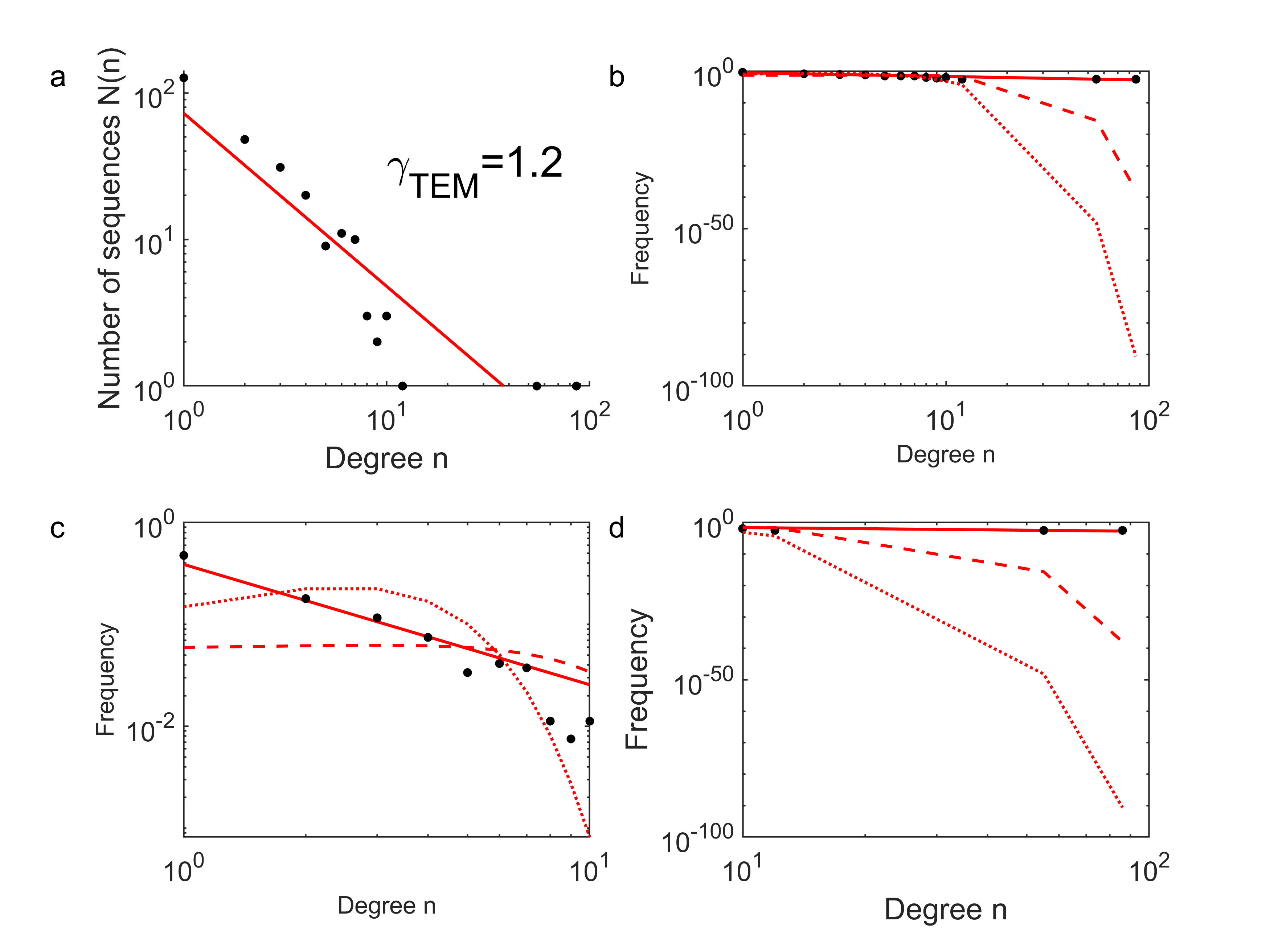

Supplement: S2 Fig — The degree distribution hints at a power law distribution with exponent γ = 1.2 (a). In addition, probability density functions were fitted for a power-law distribution (line, γ = 1.2), a Gaussian distribution (dashed line, μ = 3.0, σ = 6.4) and a Poisson distribution (dotted line, λ = 3.0) with residual sum of squares 0.01, 0.2 and 0.1, respectively (b-d). (TIF) [file pone.0200815.s004.tif]

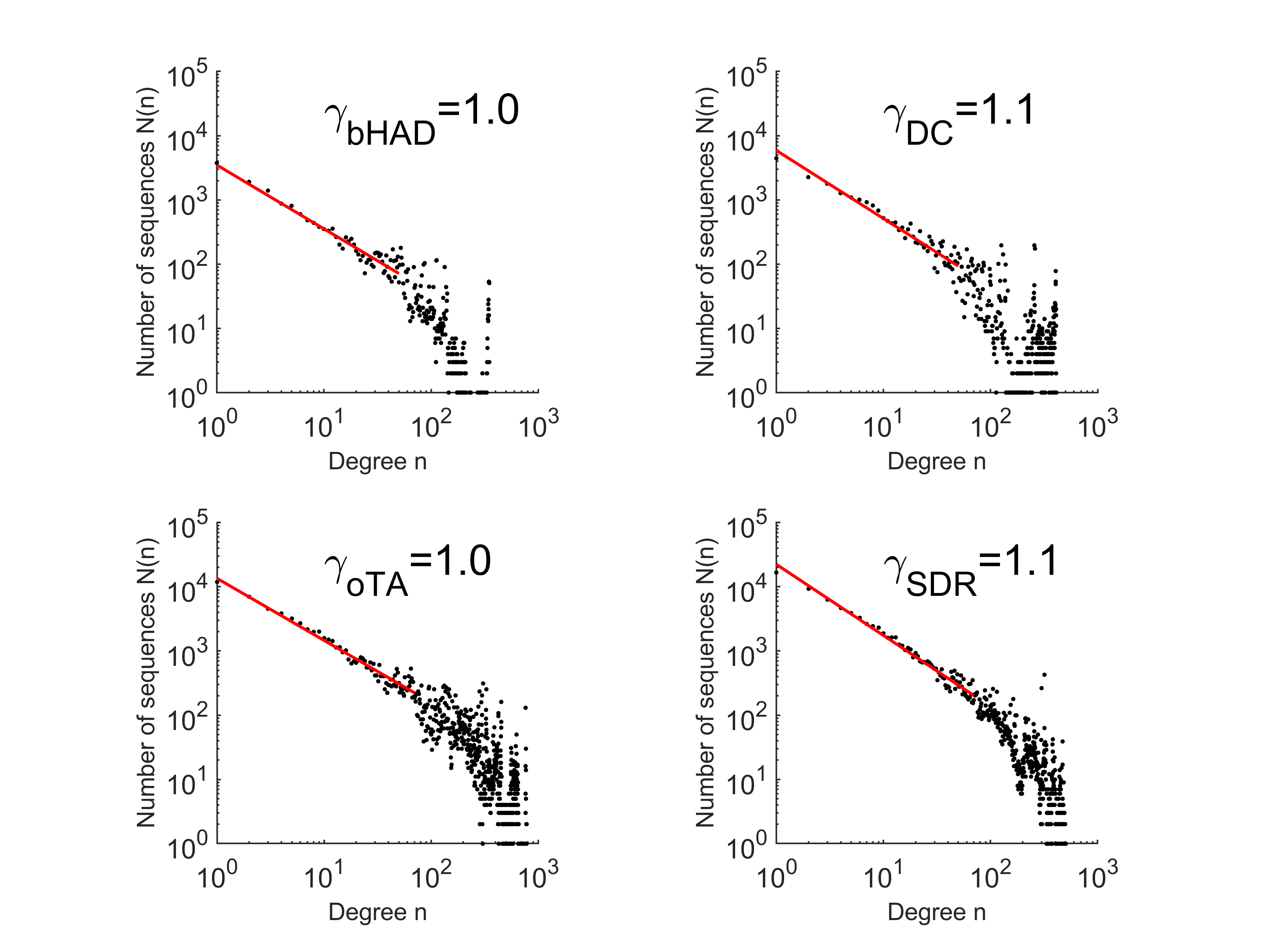

Supplement: S3 Fig — Linear regression was performed for degrees ≤ 50 (bHAD, DC) or ≤ 70 (oTA, SDR). (TIF) [file pone.0200815.s005.tif]

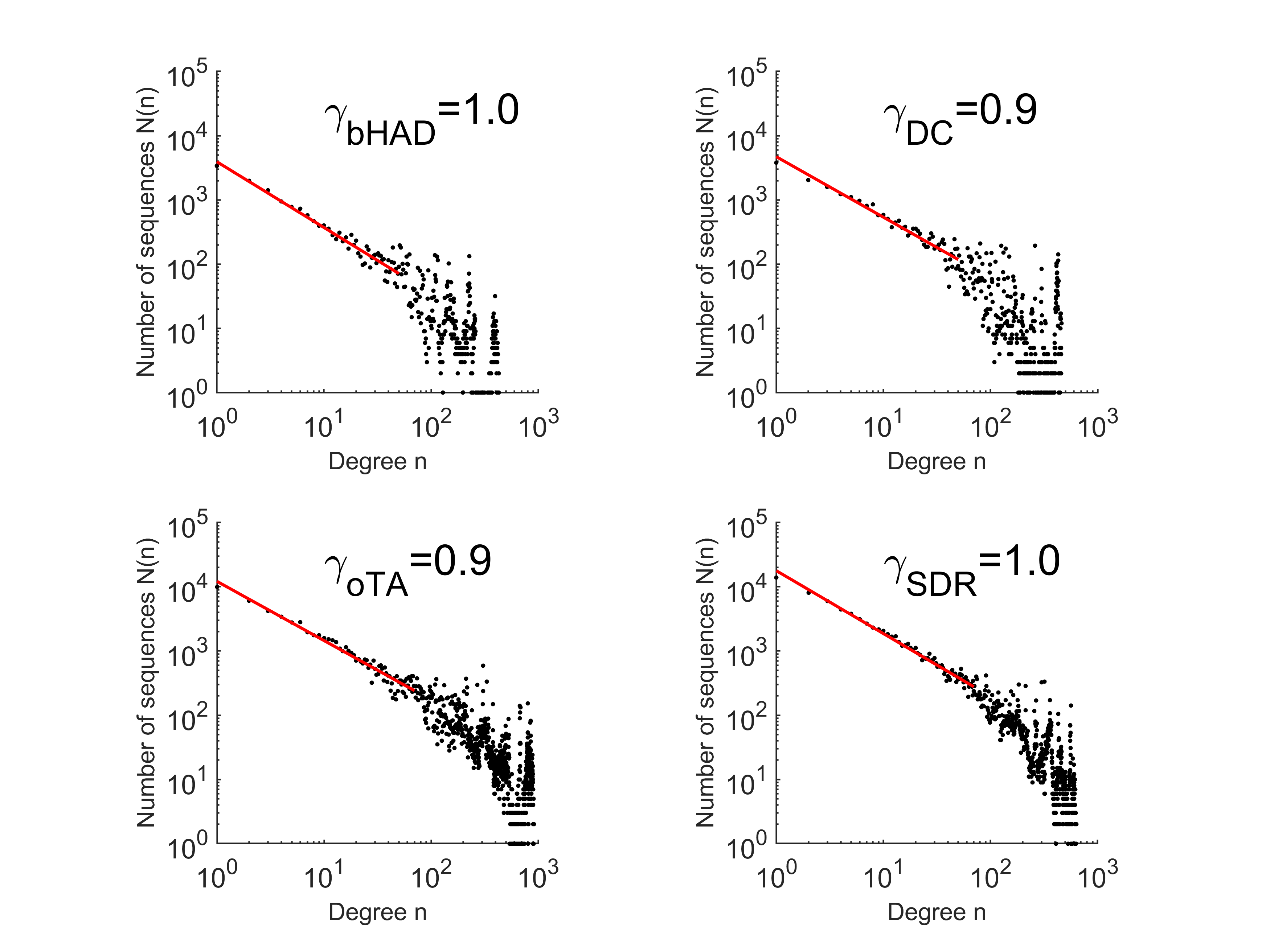

Supplement: S4 Fig — Linear regression was performed for degrees ≤ 50 (bHAD, DC) or ≤ 70 (oTA, SDR). (TIF) [file pone.0200815.s006.tif]

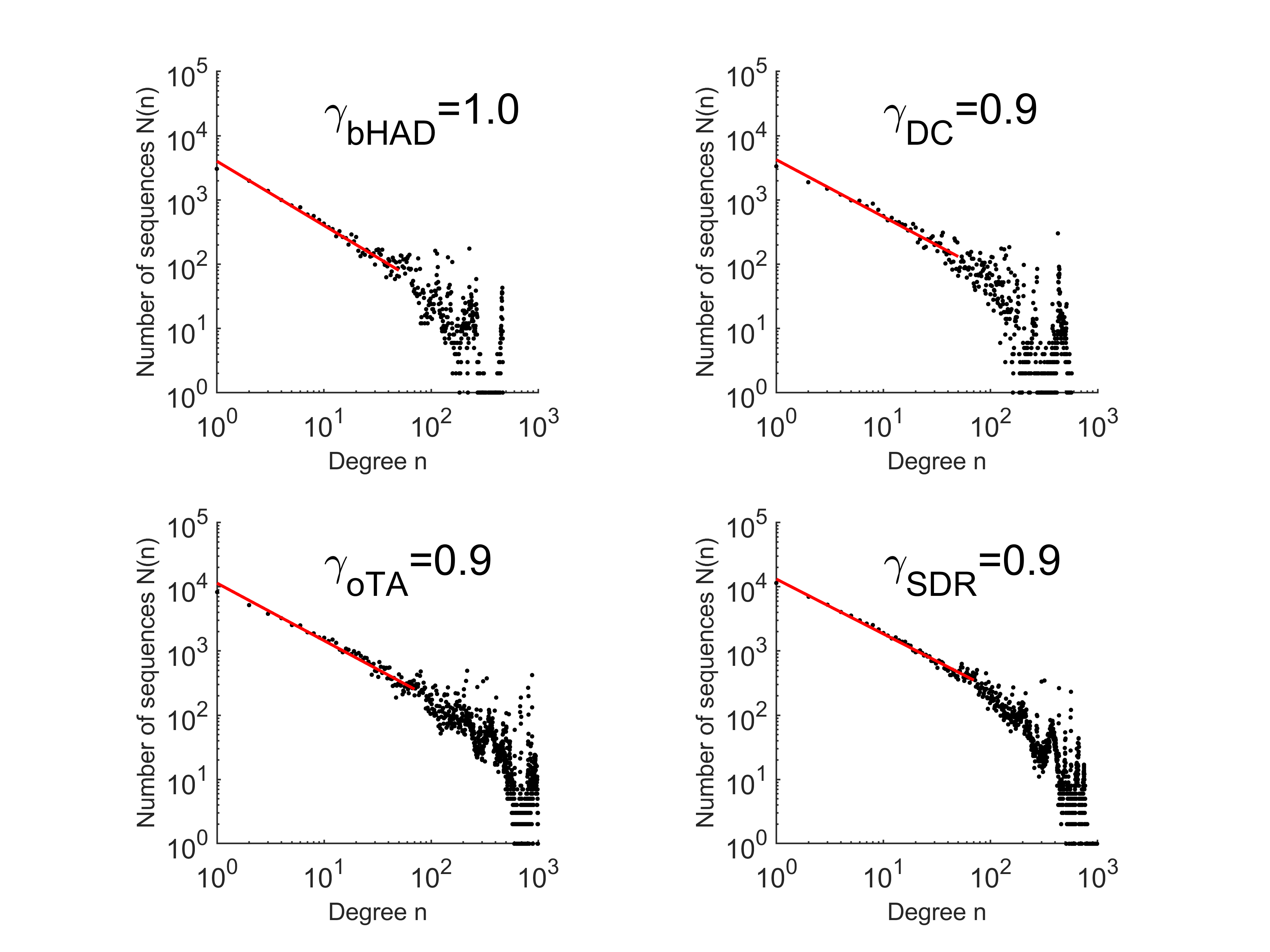

Supplement: S5 Fig — Linear regression was performed for degrees ≤ 50 (bHAD, DC) or ≤ 70 (oTA, SDR). (TIF) [file pone.0200815.s007.tif]

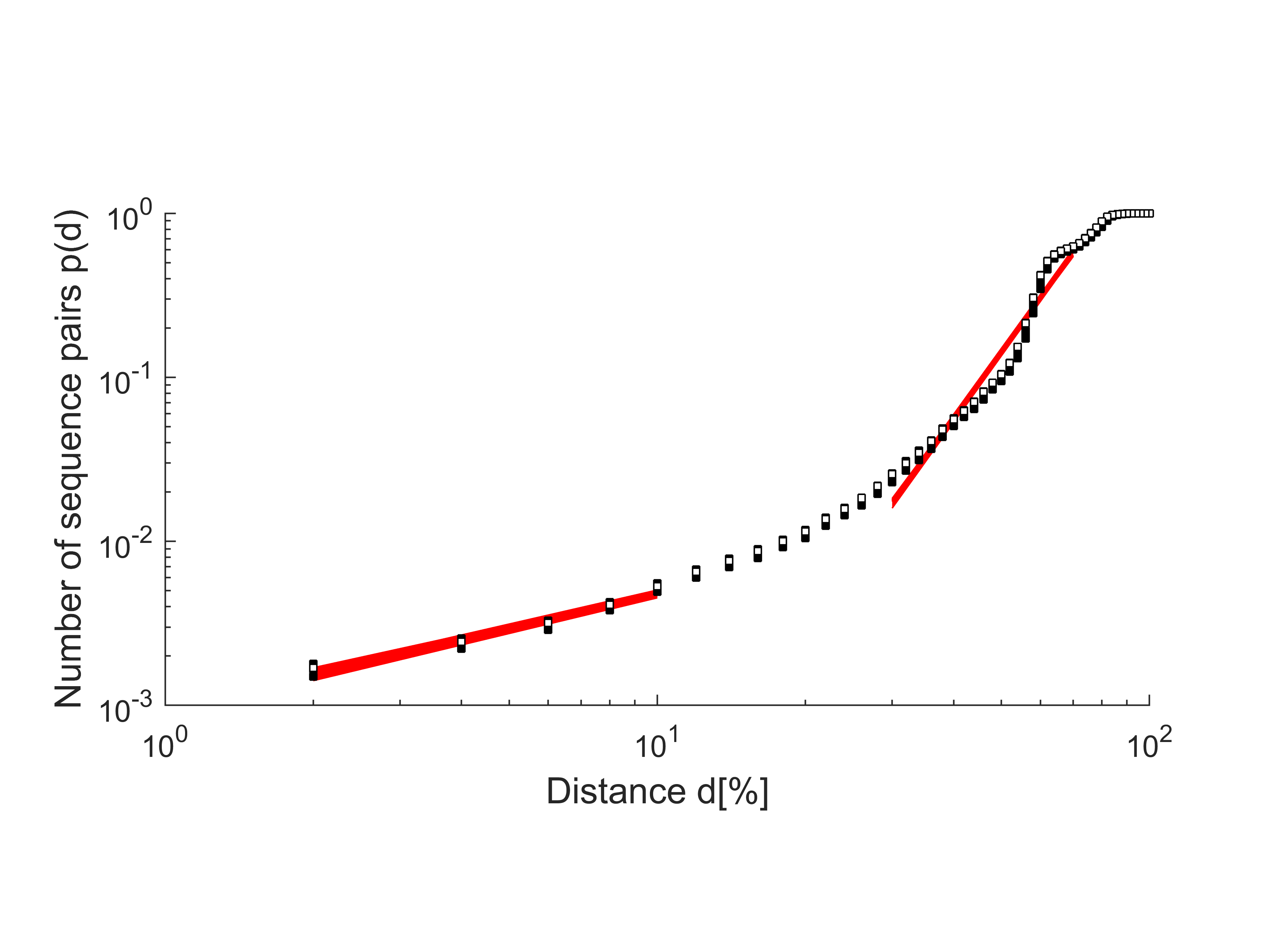

Supplement: S6 Fig — The areas marked in red correspond to the linear approximations from Fig 5. (TIF) [file pone.0200815.s008.tif]

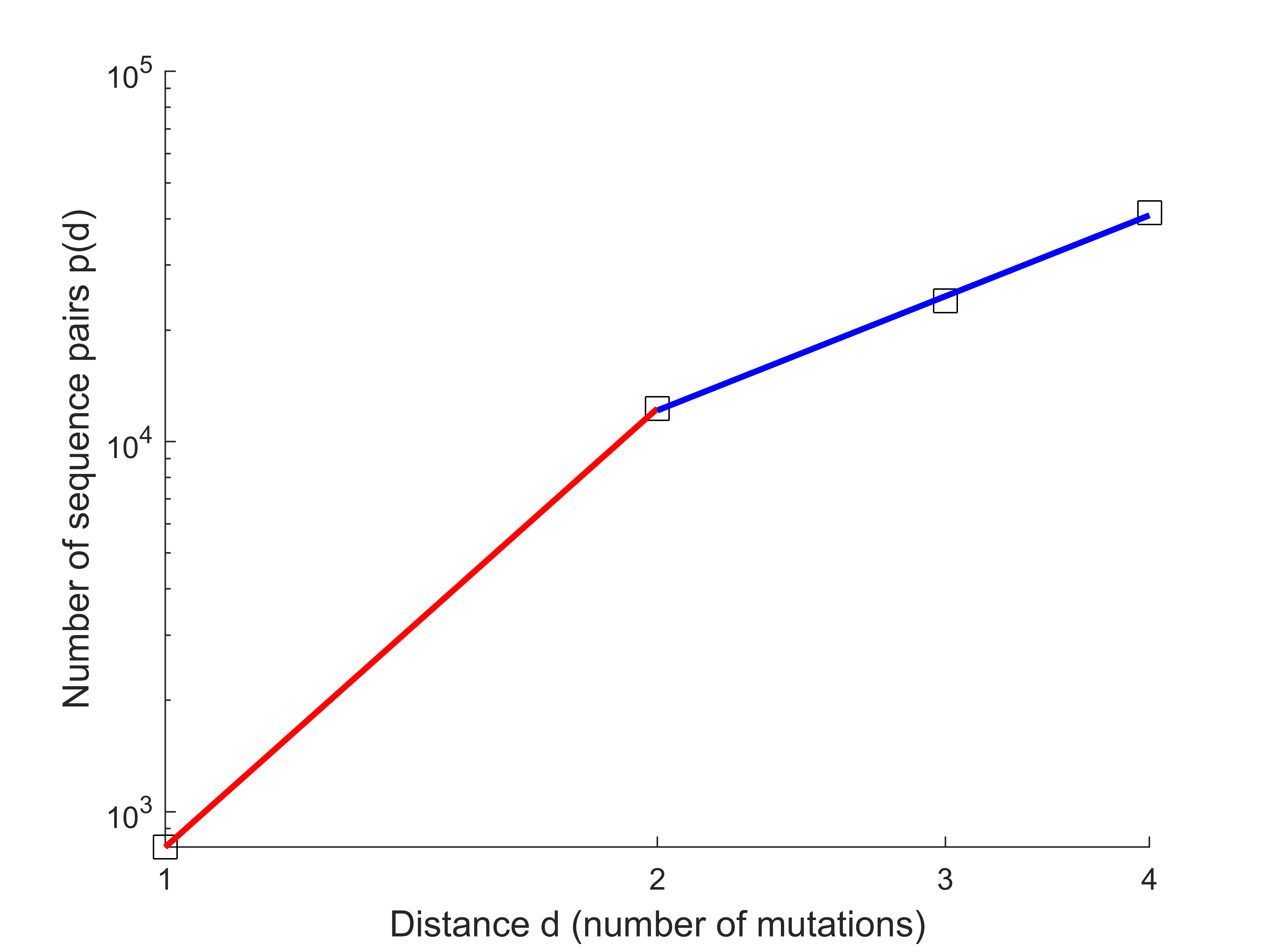

Supplement: S7 Fig — Linear fits are shown for d = 1,2 (red line) and for d = 2,3,4 point mutations (blue line). (TIF) [file pone.0200815.s009.tif]
